# Referral Patterns and Diagnostic Yield of Lung Scintigraphy in the Diagnosis of Acute Pulmonary Embolism

**DOI:** 10.1155/2017/1623868

**Published:** 2017-04-11

**Authors:** Matthieu Pelletier-Galarneau, Erik Zannier, Lionel S. Zuckier, Gregoire Le Gal

**Affiliations:** ^1^The Ottawa Hospital, Division of Nuclear Medicine, 501 Smyth Road, Ottawa, ON, Canada K1H 8L6; ^2^Department of Medicine, Ottawa Hospital Research Institute, University of Ottawa, 501 Smyth Road, Ottawa, ON, Canada K1H 8L6

## Abstract

*Introduction*. The purpose of this study is to assess referral patterns and the yield of ventilation-perfusion (V/Q) scintigraphy in patients referred for acute pulmonary embolism (PE).* Methods*. We retrospectively reviewed the charts of all patients who underwent V/Q studies between April 1, 2008, and March 31, 2010. Patients were subdivided into 4 groups based on their referral source: emergency department (ED), hospital inpatient ward, outpatient thrombosis clinic, and all other outpatient sources.* Results*. A total of 1008 patients underwent V/Q scintigraphy to exclude acute PE. The number of ED, inpatient, thrombosis clinic, and outpatient studies was 43 (4.3%), 288 (28.6%), 351 (34.8%), and 326 (32.3%). Proportion of patients with contrast contraindication varied significantly among the different groups. Of the 1,008 studies, 331 (32.8%) were interpreted as normal, 408 (40.5%) as low, 158 (15.7%) as intermediate, and 111 (11.0%) as high probability for PE. 68 (6.7%) patients underwent CTPA within 2 weeks following V/Q.* Conclusion*. The rate of nondiagnostic studies is lower than that reported in previously published data, with a relatively low rate of intermediate probability studies. Only a small fraction of patients undergoing a V/Q scan will require a CTPA.

## 1. Introduction

Pulmonary embolism (PE) is a relatively common condition affecting patients of all age groups, usually arising from thrombi developing in the lower extremity deep venous system [1]. Delayed diagnosis of PE can lead to serious medical complications and death [2]. Unfortunately, patients with acute pulmonary embolism tend to have nonspecific signs and symptoms making clinical diagnosis challenging [3]. Several noninvasive diagnostic tools are available to evaluate patients with suspected acute pulmonary embolism, including lower extremity ultrasound, D-dimer titers, CT pulmonary angiography (CTPA), and perfusion-ventilation (V/Q) scintigraphy.

In current practice, CTPA is often the first line of imaging. It is readily available and can also identify alternate causes of chest pain and/or dyspnea. However, CTPA has some disadvantages compared to V/Q scintigraphy, including administration of iodine-containing contrast and a higher radiation exposure to breast tissue [4]. Therefore, lung scintigraphy is often preferred in young females, pregnancy, and patients with renal failure and contrast allergy [5, 6]. Furthermore, because increased use of CTPA has been associated with an increase in the number of PE diagnoses without a corresponding decrease in mortality, it is thought that CTPA overdiagnoses cases of PE that are not clinically relevant [7, 8]. Finally, increased use of CTPA may be associated with detection of incidental findings requiring further investigation, often leading to unnecessary and low-yield follow-up studies [9].

Recently, Costa et al. retrospectively reviewed patients referred for CTPA at The Ottawa Hospital (TOH) with suspicion of acute PE [10]. They reviewed referral patterns and yield of CTPA performed. The purpose of this study is to assess referral patterns and the yield of V/Q scintigraphy at the same institution during the same time period, thereby facilitating comparison of these two modalities. 

## 2. Materials and Methods

### 2.1. Patient Population

This study was conducted in a tertiary care teaching hospital in Canada. The electronic charts of all patients who underwent V/Q studies between April 1, 2008, and March 31, 2010, were reviewed. Patients referred to rule out acute PE were included, while patients referred for assessment of chronic pulmonary embolism, for evaluation of chronic pulmonary hypertension, to obtain a baseline study following prior PE, and those with incomplete imaging were excluded.

The following information was retrieved when available: patient age and gender, imaging date and time, pregnancy status, referring physician, patient location, V/Q study interpretation, CTPA in the 2 weeks prior to and following V/Q study, presence of renal failure or contrast allergy, history of chronic lung disease, and D-dimer titer. Patients were subdivided into 4 groups based on their referral source: emergency department (ED), hospital inpatient ward (INPT), outpatient thrombosis clinic (TCLINIC), and all other outpatient sources (OUTPT).

### 2.2. V/Q Scintigraphy

Planar V/Q scintigraphy was performed according to standard protocol [11]. Briefly, 6 planar ventilation images (anterior, posterior, left anterior oblique, right anterior oblique, left posterior oblique, and right posterior oblique) were obtained following inhalation of 18–37 MBq (0.5–1 mCi) of ^99m^Tc-Technegas. Analogous planar perfusion images were then obtained following intravenous injection of 250,000–500,000 particles of macroaggregated albumin (MAA) labeled with 185–370 MBq (5–10 mCi) of ^99m^Tc. For pregnant patients and patients with known pulmonary hypertension, the amount of MAA particles was reduced by approximately half. Studies were interpreted by board certified nuclear medicine specialists using the modified PIOPED interpretation scheme [12]. Reports from the original interpretation were retrieved from the electronic record.

### 2.3. Statistical Analyses

Statistical analyses were performed using GraphPad Prism v7.00. Mean age of patient groups was compared using one-way ANOVA. Comparison of group characteristics was performed using the Chi-square tests. Post hoc analyses were performed using Bonferroni correction for multiple comparisons. This study was approved by The Ottawa Hospital Research Ethics Board and the requirement for patient consent was waived.

## 3. Results

**Figure 1:**
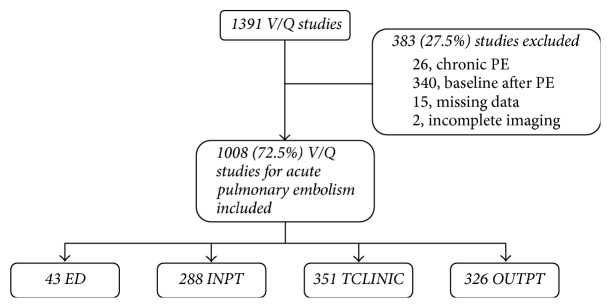
Flowchart. ED: emergency department, INPT: inpatient, OUTPT: outpatient, PE: pulmonary embolism, TCLINIC: thrombosis clinic, and V/Q: ventilation-perfusion.

A total of 1,391 V/Q studies were performed between April 2008 and March 2010 (Figure 1). Of these, 366 (26.3%) were excluded because they were performed for reasons other than to rule out an acute PE, including assessment of chronic PE and/or chronic pulmonary hypertension (26 patients) or acquisition of a baseline study following and prior to PE (340 patients). Fifteen patients (1.1%) were excluded because information could not be retrieved while 2 further patients (0.1%) were excluded due to incomplete imaging (absent ventilation or perfusion images). The number of ED, INPT, TCLINIC, and OUTPT studies was 43 (4.3%), 288 (28.6%), 351 (34.8%), and 326 (32.3%), respectively. Linear regression of the number of V/Q studies per month versus time yielded a slope not significantly different from 0 (*p* = 0.17), indicating that the rate of V/Q studies performed during the 2-year period remained stable.

### 3.1. Patient Groups

Demographic information for each group is presented in Table 1. The average age of the 4 groups differed significantly (*p*_ANOVA_ < 0.0001) and was significantly greater in the INPT group compared to all other groups (*p* < 0.01) and in the OUTPT group compared to TCLINIC (*p* < 0.001). Proportion of females varied in the different groups (*p* = 0.0012), with a statistically significant greater proportion of females in the TCLINIC compared to INPT groups (*p* = 0.021). Proportion of pregnant females also differed within the females of each group (*p* = 0.0024), with the highest proportion observed in the TCLINIC group, and a significantly greater proportion of pregnant females noted in the TCLINIC compared to the OUTPT group (*p* = 0.029). Proportion of patients with contraindication to contrast, defined as presence of contrast allergy and/or renal failure, varied significantly among the different groups (*p* < 0.001), with a lower proportion of contrast contraindications in the TCLINIC compared to all other groups (*p* < 0.001). Additionally, the proportion of patients with contraindication to contrast was also greater in the INPT compared to the OUTPT groups (*p* < 0.001).

### 3.2. V/Q Interpretation

Of the 1,008 studies, 331 (32.8%) were interpreted as normal, 408 (40.5%) as low probability, 158 (15.7%) as intermediate probability, and 111 (11.0%) as high probability for PE.  Table 2 presents the distribution of V/Q study interpretations for all patient groups; these were significantly different amongst the different groups (*p* < 0.0001). Proportion of intermediate studies was significantly greater in the INPT group (24.7%) compared to the TCLINIC and OUTPT groups (*p* < 0.001). Proportion of high probability interpretation was highest in the TCLINIC group, reaching 17.1%, and was significantly lower in the OUTPT group compared to the INPT (*p* = 0.0054) and TCLINIC (*p* < 0.001) groups.

### 3.3. Other Imaging

68/1008 (6.7%) patients underwent CTPA within 2 weeks following V/Q scintigraphy (Table 3). The overall proportion of patients who underwent subsequent CTPA did not differ significantly between the referral groups (*p* = 0.087) but did vary significantly between the V/Q interpretation subgroups (*p* < 0.0001). Overall proportion of patients who underwent subsequent CTPA ranged from less than 1% in patients with a normal V/Q scan to 21.5% in patients with an intermediate probability V/Q scan. It was significantly lower when the V/Q was interpreted as normal compared to intermediate and high probability (*p* < 0.01) and significantly lower when the V/Q was interpreted as low probability compared to intermediate probability (*p* < 0.001).

### 3.4. Subgroups Analyses

A total of 98 patients had history of chronic lung disease. Among these patients, interpretations were normal in 5 (5.1%), low probability in 57 (58.2%), intermediate in 26 (26.5%), and high probability in 10 (10.2%). The proportion of intermediate V/Q scintigraphy was greater in patients with chronic lung disease compared to all other patients (26.5% versus 14.5%, *p* = 0.0019).

A total of 74 patients were pregnant, representing 11.6% of all females. Proportion of pregnant females per group is presented in Table 1. V/Q scintigraphy interpretation and comorbidities of pregnant patients versus all other patients are presented in Table 4. V/Q scintigraphy interpretation was significantly different between the pregnant and nonpregnant patients (*p* < 0.0001), with a greater proportion of normal studies in pregnant patients (*p* < 0.0001). In the pregnant patients, prevalence of chronic lung disease (*p* = 0.012), chronic renal failure (*p* < 0.0001), and chronic heart failure (*p* = 0.0063) were statistically lower compared to nonpregnant patients. For pregnant patients, prevalence of pulmonary hypertension (*p* = 0.052) and contrast allergy (*p* = 0.096) was lower than in nonpregnant patients but did not quite reach 95% confidence levels. Ten (13.5%) of the 74 pregnant patients received anticoagulation therapy within 3 months following the V/Q scintigraphy, 4 for reasons other than PE (DVT or history of spontaneous abortion) and 6 to treat PE, 5 with high probability, and 1 with intermediate probability V/Q study results. Three (4.1%) of the 74 pregnant patients underwent CTPA in the 2 weeks following V/Q scan. Two had intermediate probability and 1 had low probability V/Q results; all 3 had positive D-dimer titers. Final diagnosis was pneumonia in one patient and lung fibrosis in another, while a final diagnosis was not reached for the third patient. None of the 3 patients received anticoagulation therapy.

## 4. Discussion

In this study, we report on the referral patterns and yield of V/Q scan over a two-year period in our institution. At our institution, the number of V/Q scans is much lower than the number of CTPAs. Over the identical time period, 1,677 and 1,894 CTPA studies were performed from the ED and INPT groups [10] as compared to 43 and 288 V/Q studies, respectively, corresponding to a 1 : 12 V/Q to CTPA ratio. The smaller number of V/Q scans requested from the ED follows a trend over the last decade of increasing use of CT imaging to rule out PE [13]. As opposed to planar V/Q scintigraphy, CTPA offers a binary interpretation which is considered convenient by some, especially in the ED setting. Indeed, the significantly higher rate of contrast contraindications in the patients referred from ED and INPT indicates that V/Q scan is often requested when CTPA cannot be performed. Rate of V/Q utilization also likely related to the local practices at our center where stable patients are often managed as outpatients and directly referred to the thrombosis clinic for diagnostic management, whereas unstable patients, or patients with comorbidities, are often directly referred for imaging from the emergency department. Under these circumstances, imaging is usually requested by the clinic physician and not the ED physician.

Some findings in this study deserve further comment. TCLINIC was the largest source of patient referrals, and these patients were overall younger and had less comorbidities, while a large proportion of INPT patients had contraindications to CTPA. The overall proportion of intermediate probability V/Q studies was 15.7%. This is significantly lower than the 44% proportion noted in the PIOPED I cohort [14] but is comparable to the percentage of indeterminate or limited results at CTPA, which is reported to be between 5% and 25% [15–18]. The yield of V/Q scintigraphy was different among the different groups studied. The proportion of high probability studies was greatest in patients referred from the thrombosis clinics while the proportion of normal/low probability studies was highest in the outpatient population. The proportion of high probability studies from the ED and inpatients was approximately 10%, compared to the positive CTPA rate of 15% obtained over the same time period and population at our institution [10]. The differences in V/Q yield among the different groups could be in part related to the differences in referral patterns. Indeed, the patients referred in the ED and INPT groups had higher rates of comorbidities, including renal failure, and the INPT group had the highest mean age and highest proportion of patients with CHF and chronic pulmonary disease. Furthermore, an elevated rate of high probability results in the TCLINIC group could also be in part related to better patient selection.

The vast majority of patients (93.3%) who underwent V/Q scintigraphy did not require further investigation with CTPA. This highlights the fact that although V/Q scintigraphy is often criticized for a high rate of inconclusive results, further imaging with CTPA is usually not necessary when V/Q scan is combined with pretest probability assessment, D-dimer titers, and venous compression ultrasonography (CUS) of the lower leg. Previous studies have shown that patients with inconclusive V/Q scintigraphy can be safely managed with serial leg CUS [7]. When the V/Q study was normal or low probability, CTPA was performed only in 3.3% of patients, underlining appreciation of the high negative predictive value of V/Q scintigraphy to exclude PE. Not surprisingly, the rate of subsequent investigation with CTPA was the highest when probability was intermediate on V/Q scintigraphy, reaching 21.5%. Nevertheless, the relatively low prevalence of intermediate V/Q results explains the overall low rates of CTPA performed following V/Q scintigraphy.

The presence of underlying chronic lung disease increases the number of intermediate V/Q results. Of the 98 patients with chronic lung disease, 26.5% had an intermediate V/Q result compared to 14.5% for all other patients. Although the proportion of intermediate V/Q result is greater in patients with underlying lung disease, it remains relatively low and below the frequently quoted 50–80% [19, 20]. These results suggest that, even in the presence of chronic lung disease, V/Q scintigraphy could be employed as a first-line modality in patients with underlying chronic lung disease, even in the absence of any contraindication to contrast.

Some authors have recommended against the use of V/Q scintigraphy in pregnant patients due to a relatively elevated proportion of intermediate V/Q results, potentially leading to additional investigation with CTPA. In our sample, 6.8% of pregnant patients had intermediate V/Q results which compare favorably to the 33% suboptimal CTPA rate reported in pregnant patients [21]. Furthermore, in our sample, only 4.1% of pregnant patients underwent subsequent CTPA. The low rate of intermediate study results and need for additional imaging support current guidelines recommending the use of V/Q scintigraphy in the population of pregnant patients [22].

Amongst our study's limitations, this was a retrospective analysis. Although we were able to identify all patients who underwent V/Q scintigraphy in our institution, and to select only those in whom the V/Q scan was requested for the workup of a suspected PE, we could not conclusively discern diagnostic algorithms or why one test was chosen over another in each individual. Our study period is also somewhat remote (2008–2010) and might not reflect most recent practice; however, this interval was chosen to align with a similar study performed on the use of CTPA at our institution and our particular practice has remained constant since this period.

In summary, we have shown that the rate of nondiagnostic studies is lower than that reported in previously published data, with a relatively high rate of high probability studies and a low rate of intermediate probability studies. Only a small fraction of patients undergoing a V/Q scan will require a CTPA to complete their investigation. This observation also holds true in subgroups of pregnant patients and those with chronic lung disease that we have studied. V/Q scintigraphy remains a useful diagnostic tool in the context of acute PE, particularly for patients without underlying lung pathologies/abnormalities.

## Figures and Tables

**Table 1:** Summary of patient demographics and V/Q scintigraphy results for different referral sources.

	ED	INPT	TCLINIC	OUTPT
*N* V/Q studies	43	288	351	326
*N* 1st year	20	142	189	185
*N* 2nd year	23	146	162	141
*N* weekdays (%)	33 (76.6)	257 (89.2)	279 (79.5)	311 (95.4)
*N* weekends (%)	10 (23.3)	31 (10.8)	72 (20.5)	15 (4.6)
*N* female (%)	26 (60.5)	155 (53.8)	239 (68.1)	216 (66.3)
*N* pregnant (% of females)	2 (7.7)	16 (10.3)	42 (17.6)	14 (6.5)
Mean age ± SD	53.9 ± 22.6	64.7 ± 19.3	46.3 ± 19.2	58.5 ± 18.9
Age range	20–89	17–99	18–93	18–96
Contrast contraindication	19 (44.2)	172 (59.7)	43 (12.3)	82 (25.2)
*N* renal failure (%)	18 (41.9)	158 (54.9)	34 (9.7)	73 (22.4)
*N* contrast allergy (%)	4 (9.3)	28 (7.3)	12 (3.4)	13 (4.0)
*N* pulmonary hypertension (%)	0 (0.0)	23 (8.0)	1 (0.3)	45 (13.8)
*N* chronic lung disease (%)	1 (2.3)	55 (19.1)	16 (4.6)	26 (8.0)
*N* chronic heart failure (%)	3 (7.0)	58 (20.1)	3 (0.9)	23 (7.1)

**Table 2:** V/Q study results per group. Percentages refer to interpretation groups within each referral source.

Interpretation	ED	INPT	TCLINIC	OUTPT
*N* normal (%)	19 (44.2)	36 (12.5)	141 (40.2)	135 (41.4)
*N* low (%)	14 (32.6)	148 (51.4)	109 (31.1)	137 (42.0)
*N* intermediate (%)	6 (14.0)	71 (24.7)	41 (11.7)	40 (12.3)
*N* high (%)	4 (9.3)	33 (11.5)	60 (17.1)	14 (4.3)

**Table 3:** Proportion of patients who underwent computed tomography pulmonary angiography (CTPA) within 2 weeks following V/Q scintigraphy for each patient group and V/Q interpretation.

	ED	INPT	TCLINIC	OUTPT	Overall
Normal (%)	0/19 (0)	0/36 (0)	0/141 (0)	3/135 (2.2)	3/331 (0.9)
Low (%)	1/14 (7.1)	6/148 (4.1)	6/109 (5.5)	8/137 (5.8)	21/408 (5.1)
Intermediate (%)	0/6 (0)	18/71 (25.4)	5/41 (12.2)	11/40 (27.5)	34/158 (21.5)
High (%)	0/4 (0)	3/33 (9.1)	6/60 (10.0)	1/14 (7.1)	10/111 (9.0)

Overall	1/43 (2.3)	27/288 (9.4)	17/351 (4.8)	23/326 (7.1)	68/1008 (6.7)

**Table 4:** Summary of V/Q scintigraphy results and comorbidities in pregnant versus nonpregnant patients.

	Pregnant	Nonpregnant
*N*	74	934
V/Q interpretation		
*N* normal (%)	46 (62.2)	285 (30.5)
*N* low (%)	18 (24.3)	390 (41.8)
*N* intermediate (%)	5 (6.8)	153 (16.4)
*N* high (%)	5 (6.8)	106 (11.3)
*N* contrast contraindication (%)	2 (2.7)	316 (33.8)
*N* renal failure (%)	1 (1.4)	282 (30.2)
*N* contrast allergy (%)	1 (1.4)	56 (6.0)
*N* pulmonary hypertension (%)	1 (1.4)	68 (7.3)
*N* chronic lung disease (%)	1 (1.4)	97 (10.4)
*N* chronic heart failure (%)	0 (0)	86 (9.2)
